# Treatment of Grade 3 and 4 Osteoarthritis with Intraoperatively Separated Adipose Tissue-Derived Stromal Vascular Fraction: A Comparative Case Series

**DOI:** 10.3390/cells9092096

**Published:** 2020-09-14

**Authors:** Denis Simunec, Honey Salari, Juliane Meyer

**Affiliations:** 1 Plastic, Aesthetic Hand- & Reconstructive Surgery, Marien Hospital Soest, 59494 Soest, Germany; info@revitcells.com (D.S.); honeysalari@gmail.com (H.S.); 2 Medical Affairs, Human Med AG, 19061 Schwerin, Germany

**Keywords:** stromal vascular fraction (SVF), therapeutics, osteoarthritis (OA), adipose tissue-derived stem/stromal cells (ASCs), Q-graft^®^

## Abstract

Osteoarthritis (OA) is the most common form of arthritis of the joints. The stromal vascular fraction (SVF) is a regenerative cell population that can be isolated from adipose tissue. It is the immunomodulatory properties of the stromal vascular fraction that make it a promising candidate for the regenerative treatment of OA. Patients with grade 3 and 4 osteoarthritis were treated with the stromal vascular fraction with and without platelet-rich plasma (PRP) and followed up on their Knee Injury and Osteoarthritis Outcome Score (KOOS) score for 12 months, with MRI and subjective evaluation of the procedure. Magnetic resonance imaging (MRI) revealed a widening of the joint space, a restructuring of the cartilage, and an alleviation of effusions in the treated joints. In three of the four treatment groups, a substantial improvement of the KOOS scores was documented at the 12-month follow-up time point. According to the subjective evaluation, 67% of the patients were satisfied or very satisfied with the procedure and would recommend it to others. No serious adverse events or unwanted side effects related to the SVF treatment were observed or reported. Prior to an invasive artificial joint replacement, the treatment of arthritic knee joints with the intraarticular injection of autologous adipose tissue-derived SVF should be considered a regenerative treatment option.

## 1. Introduction

Osteoarthritis (OA) is the most common form of arthritis and most frequently affects the knees, hips, and small hand joints [1]. Osteoarthritis can cause loss of cartilage, sclerosis of the subchondral bone, and synovitis. First and foremost, it causes debilitating pain [1]. The progression of OA involves continuous inflammation of the joints caused by the production of reactive oxygen species, cytokines, chemokines, and other proinflammatory products by the affected chondrocytes [2]. Conservative treatments of OA range from nonpharmacological (e.g., weight loss, physical therapy, and exercise) through pharmacological (e.g., nonsteroidal anti-inflammatory drugs or glucocorticoid injections) to surgical treatments (e.g., osteochondral grafts or microfracture), the last option for most patients being a total joint replacement [3]. None of those treatments can ultimately stop the progression of the degeneration of the joint tissues, let alone regenerate them [4]. It is thus one of the challenges of clinical research to find a treatment for OA that can not only mitigate the symptoms of OA but sustainably stop the progression of the disease and reverse chondral tissue damages.

Since the discovery and characterization of the multipotent stem cell population in adipose tissue by Patricia Zuk et al. in 2002 [5], the regenerative properties of the stromal vascular fraction (SVF) have been investigated in multiple preclinical and clinical models [6,7,8,9,10]. The SVF is a cell population that can be conjointly retrieved by the process of enzymatic dissociation filtration and centrifugation of adipose tissue [11]. In particular, it is the immunomodulatory properties of the SVF that make it a promising candidate for the regenerative treatment of OA [12,13]. The adipose tissue-derived mesenchymal stem/stromal cells (ASCs) in the SVF secrete several anti-inflammatory substances like IL-1RA, nitric oxide, TGFβ1, SDF-1, and LL37, among others. These alleviate the inflammatory state in the diseased joint (reviewed in [14]). Whether the injected cells of the SVF in fact contribute to the actual regeneration of the cartilaginous tissue remains yet to be fully elucidated. While some studies reported a quantifiable regeneration of cartilage [8,15,16], others did not observe any changes [17,18]. Even if the de novo formation of the cartilaginous tissue can be documented, the following questions remain: What type of cartilage has been formed? Has the new tissue been formed by the injected cells that have homed in on the damaged parts of the joint and differentiated into tissue-specific cells, or has the alleviation of the inflamed state enabled the body to reactivate its own repair mechanisms? This is a matter of ongoing research.

Another open question is the optimal layout of the actual therapeutic approach, meaning the dose of cells that is most appropriate to treat an affected joint and the mode of administration. Currently, pursued strategies differ widely in their designs. Some include application with and without visual guidance [8,19], with and without the addition of platelet-rich plasma (PRP) to the SVF [17,19], or with or without a predefined dosage of cells [20,21]. To this date, there has been no clinical consensus on the questions listed because the database available to draw such conclusions is not concise enough.

In the present work, we evaluated the clinical follow-up data of patients that were treated with regenerative cells of the SVF. In 2017, we were the first clinic in the world to apply the Q-graft^®^ device as a novel system for the compassionate treatment of patients with knee osteoarthritis. During clinical follow-up, we documented possible adverse events and compared the progression of the OA grade between the different treatment groups. With the help of the collected data, it is the aim of this present work to demonstrate the safety of the procedure and to contribute to the clarification of some of the open questions in the field.

## 2. Materials and Methods

### 2.1. Patients

**Figure 1:**
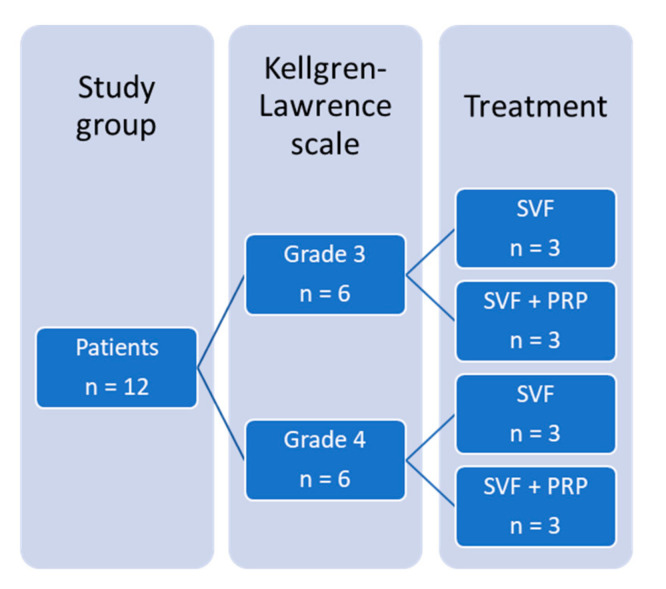
Structuring of the study group. The study group consisted of 12 patients. Six of them had OA grade 3 on the Kellgren–Lawrence scale; 6 of them had OA grade 4. Within each group, 3 patients were treated with the SVF, and 3 patients were treated with the SVF and PRP. OA, osteoarthritis; SVF, stromal vascular fraction; PRP, platelet-rich plasma.

All treatments were performed according to the principles of the Declaration of Helsinki 1996. All patients were treated in the course of compassionate treatment attempts after thoroughly informed consent. A total of 12 patients were included in this series. They were divided into four groups, separated based on the radiographic grade of OA using the Kellgren–Lawrence scale, as well as on the involvement of PRP in the treatment. The structuring of the study group can be found in Figure 1. Differences in patient demographics between the treatment groups are listed in Table 1.

### 2.2. Inclusion/Exclusion Criteria

All patients treated in our hospital for the first time with SVF cells between February 2017 and July 2018 with symptomatic Kellgren–Lawrence grade 3 and 4 OA of the knee were selected to be included in this case series regardless of age, gender, and BMI, given they were willing to participate in the case series and had filled out the forms completely. In order to be able to compare the outcomes, we excluded all patients with treatment sites other than the knee and those who had incompletely answered forms.

### 2.3. Clinical Evaluation

The grade of OA was radiographically assessed using X-ray images, graded according to the Kellgren–Lawrence scale on the first visit. The clinical follow-up time was at least 12 months. To assess the subjective outcome, all treated patients were asked to fill out the Knee Injury and Osteoarthritis Outcome Score (KOOS) (German version, by Kessler et al., 2003 [22]) surveys at certain intervals, focusing on the status preoperatively, as well as after 1, 3, 6, and 12 months postoperatively. The subjective outcome was assessed with a simple questionnaire with closed-ended questions concerning overall satisfaction and generally how recommendable the procedure was.

### 2.4. Tissue Harvesting

The adipose tissue was acquired in the operating room under sedation and pain relief or under total anesthesia with water-jet-assisted liposuction using the body-jet^®^ evo (Human Med AG, Schwerin, Germany). An amount of 50 mL of tumescent solution with the addition of vitamin C (modified tumescent solution by Simunec) was infiltrated into the harvest area of the abdomen. After a waiting period of 15 min, a maximum amount of 75 mL was obtained and collected in the cell separation system Q-graft^®^ (Human Med AG, Schwerin, Germany).

### 2.5. SVF Separation

**Figure 2:**
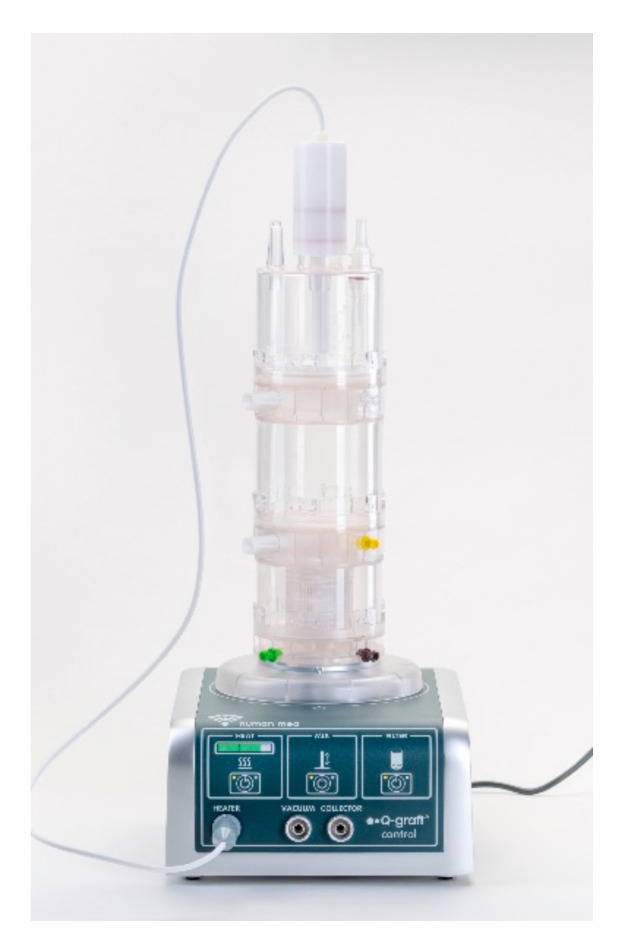
Separation system for regenerative cells from adipose tissue Q-graft^®^ (Human Med AG, Schwerin, Germany) consisting of the disposable part Q-graft^®^ collector (top) and the reusable control unit Q-graft^®^ control (bottom).

After harvesting 75 mL of tissue into the top chamber of the Q-graft^®^ collector (see Figure 2), 20 mL of a low-dose collagenase (Humanase™, Human Med AG, Schwerin, Germany) was added.

After the activation of a heating and mixing function on the Q-graft^®^ control, the tissue was digested for 45 min at 37 °C. Following the digestion period, the tissue was washed twice with cold Sterofundin and filtered into the middle chamber of the Q-graft^®^ collector. The suspension containing the separated, washed, and filtered SVF cells was extracted from the lower chamber of the Q-graft^®^ collector into a 50 mL syringe at a speed of 1 mL/sin order to avoid any damaging shear stress on the cells. This step was repeated until the entire suspension was transferred into 50 mL centrifugation tubes. The tubes were then centrifuged at room temperature for 5 min at 400× *g*. The resulting pellets were extracted from the tubes into a small volume of liquid. The resulting suspension was diluted fivefold using Sterofundin. In the case of the supplementary treatment with PRP, the resulting suspension was diluted fivefold using combined Sterofundin and PRP. An amount of 1 mL of that suspension was used for cell counting.

### 2.6. Cell Counting

Cell counting was done in duplicate measurements with the NucleoCounter^®^ NC-200™, using the “Viability and Cell Count-Aggregated Cells Assay” (ChemoMetec, Allerod, Denmark) according to the manufacturer’s instructions.

### 2.7. PRP Preparation

PRP was prepared from whole blood taken from a vein in the arm using the BRC3-Kit (Regen Lab, Munich, Germany) according to the manufacturer’s instructions.

### 2.8. Intra-Articular SVF Injection

After disinfection and dressing of the knee to be treated, local anesthesia (lidocaine, 5 mL) was applied to the capsule via a lateral access using the no-touch technique. If the operation was done under general anesthesia, local anesthesia was not required. Using an 11 mm blade, a stab incision was made at the lateral access. The knee joint was punctured with a 7 cm 20 G needle attached to a 10 mL syringe. The puncturing of the knee was done with the visual assistance of real-time X-ray (C-arm). After extraction of synovial fluid, the syringe was removed from the cannula. The cannula remained in the knee joint. The syringe with the SVF suspension was attached to the cannula, and the SVF suspension was injected into the knee joint. Between 7 and 37 mL of SVF suspension was injected (for injection volumes, please refer to Table 2). After the application of an adhesive bandage, the knee was moved manually to distribute the SVF suspension homogeneously within the joint. Final wrapping of the leg was done from distal to proximal with cotton wool and elastic bandaging.

### 2.9. Statistical Data Analysis

The numbers of donors are mentioned in the figure and table legends. Calculations and statistical analyses were carried out with Microsoft Excel Office 365 Business and GraphPad Prism 8 for Windows 64-bit version 8.4.1. The measurements for the isolated cell numbers for one single donor were done in technical duplicate. The normality of the data distribution was tested with the Shapiro–Wilk and the Anderson–Darling tests. The homogeneity of variance of the KOOS scores between the baseline and the follow-up time points was analyzed with the nonparametric Wilcoxon matched-pairs signed rank test for paired data (*p* < 0.05). The correlation of the number of cells injected, the stage of osteoarthritis, and age with the progression of the KOOS scores at the follow-up time points of 3 and 12 months was evaluated using the Pearson correlation coefficient *r*. All diagrams were created with the GraphPad software. The data displayed in graphs are means with standard deviation.

## 3. Results

In the following, the outcomes of the treatments of the 12 patients with knee osteoarthritis with freshly isolated SVF and joint injection in one operative procedure are listed. The treatments were carried out between February 2017 and July 2018. In a 1-year follow up period, objective and subjective outcome measurements of the procedures were recorded.

### 3.1. Cell Numbers for the SVF Treatment

The preparation of the SVF cells was done with a novel device for the intraoperative isolation and concentration of the SVF cells from autologous adipose tissue. In Table 2, the injection volumes per joint and the contained cell numbers, as well as the addition of PRP, are listed.

An average of 7.56 × 10^6^ cells ranging from 4.24 × 10^6^ to 17.2 × 10^6^ were injected to each knee joint. Without the addition of PRP, an average volume of 10.5 mL of SVF suspension was injected; with the addition of PRP, an average of 22.9 mL of SVF suspension was injected to each knee joint.

### 3.2. Objective Clinical Outcomes

**Figure 3:**
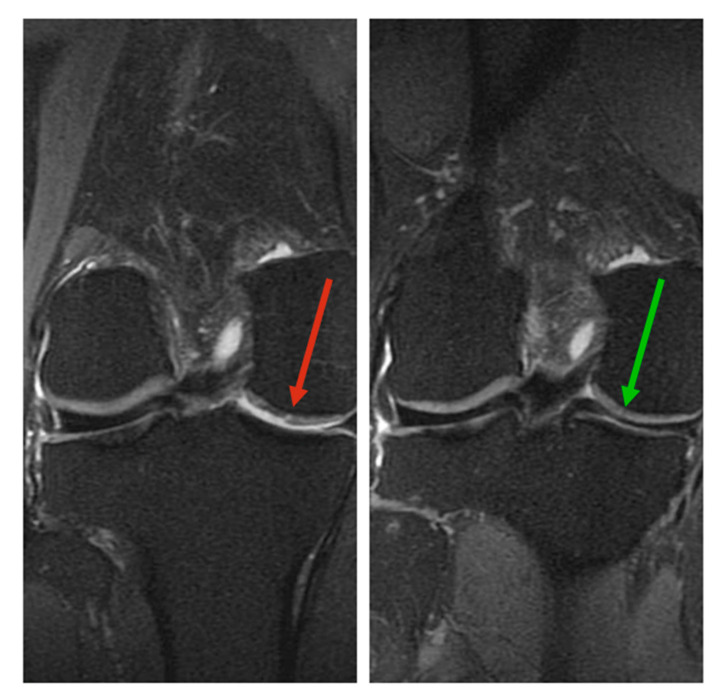
Magnetic resonance imaging (MRI) status of the right knee joint before treatment with the SVF (**left**), showing lesion in the cartilage (red arrow) and 16 months after treatment with SVF (**right**), showing potential cartilage regeneration and disappearance of the effusion (green arrow).

**Figure 4:**
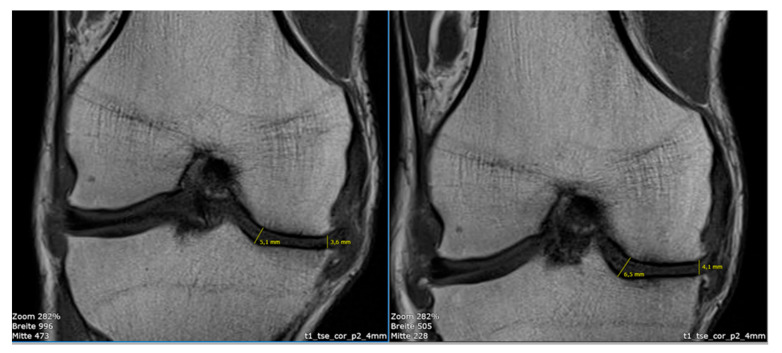
Magnetic resonance imaging (MRI) status of the right knee joint before treatment with the SVF and PRP (**left**), showing a joint space of 5.1 mm at the saddle point and 3.6 mm in the medial area. At 14 months after treatment with the SVF and PRP (**right**), the joint space was 6.5 mm at the saddle point and 4.1 mm in the medial area.

Two effects that could be observed in some patients by pre- and postoperative imaging (MRI) were a restructuring of the cartilage (Figure 3) and an increase in joint space (Figure 4).

Figure 3 shows an example of the MRI status of the right knee joint before and 16 months after the treatment with the SVF. Before the treatment, the image displays a lesion in the cartilage, as well as an effusion and an overall rather unstructured cartilage configuration. The image displaying the same joint 16 months after the SVF treatment shows areas that could potentially represent cartilage regeneration. The cartilage has a more harmonious structure overall, and the effusion can no longer be observed.

Figure 4 shows the MRI of the right knee joint of one of the patients before the treatment with the SVF and PRP and after the treatment. Before the treatment, the joint space measured 5.1 mm at the saddle point and 3.6 mm in the medial area. Fourteen months after the treatment, the joint space increased to 6.5 mm at the saddle point and to 4.1 mm in the medial area.

A change in cartilage structure or joint space was not detectable in all treated joints.

### 3.3. Progression KOOS Score Due to Treatment with SVF

During a follow-up period of 1 year, the KOOS score of the patients was evaluated at baseline, as well as 1, 3, 6, and 12 months after the procedure.

**Figure 5:**
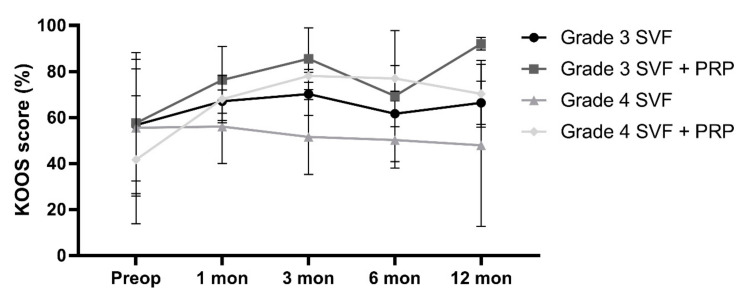
Progression of the KOOS scores of the four treatment groups at baseline as well as 1, 3, 6, and 12 months after the treatment of knee osteoarthritis with freshly isolated SVF from the adipose tissue; n = 3 for each group at each time point; KOOS, Knee injury and Osteoarthritis Outcome Score. PRP, platelet-rich plasma.

Figure 5 displays the progression of the KOOS scores of the four treatment groups over the follow-up period of 1 year.

In the group of patients with grade 3 osteoarthritis that were treated with the SVF without the addition of PRP, the average improvement of the KOOS score was 34.5% from the preoperative state to 12 months after the treatment. In the group of patients with grade 3 osteoarthritis that were treated with the SVF with the addition of PRP, the average improvement of the KOOS score was 9.7% over the same follow-up period. In the group of patients with grade 4 osteoarthritis that were treated with the SVF without the addition of PRP, the average KOOS score dropped by 7.7% from the preoperative state to 12 months after the treatment. In the group of patients with grade 4 osteoarthritis that were treated with the SVF with the addition of PRP, the average improvement of the KOOS score was 28.8% from the preoperative state to 12 months after the treatment.

### 3.4. Subjective Evaluation of the SVF Treatment by the Patients

**Figure 6:**
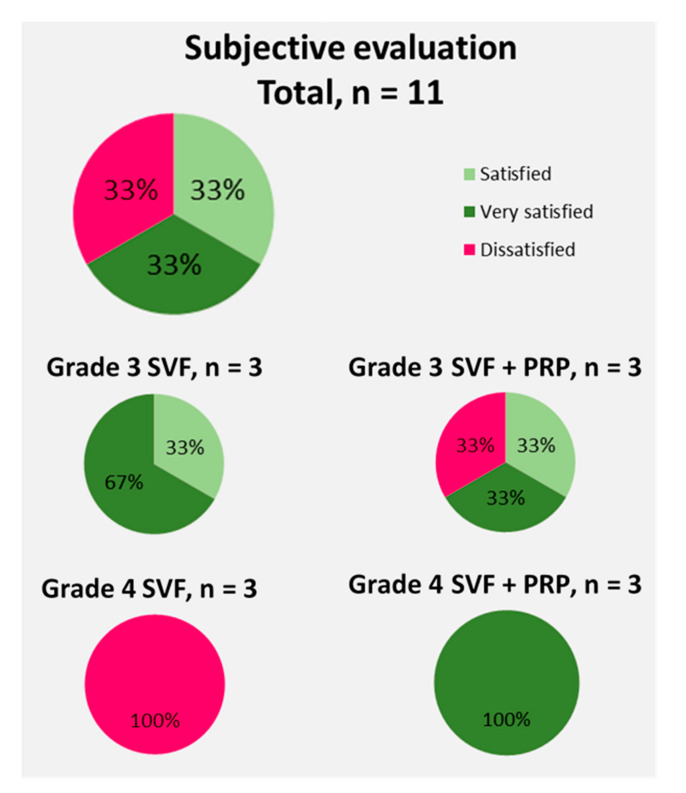
Subjective evaluation of the 11 patients on their overall satisfaction with the SVF treatment of their knee osteoarthritis with freshly isolated SVF from the adipose tissue.

**Figure 7:**
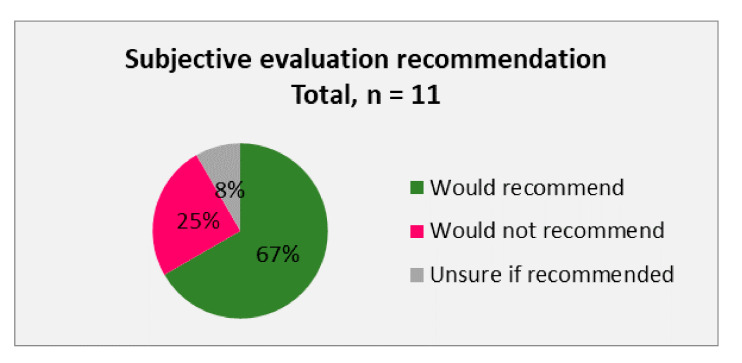
Subjective evaluation of the 11 patients on whether they would recommend the treatment of their knee osteoarthritis with freshly isolated SVF from the adipose tissue.

On an average of 8 months after the procedure, a subjective patient evaluation was done. Herein the patients were asked about their satisfaction (Figure 6) with the procedure and whether they would recommend the treatment or not (Figure 7).

Only 11 of the 12 patients could be surveyed as 1 patient had opted not to answer the questionnaire for subjective evaluation. Overall, 67% of the patients queried were satisfied or very satisfied with the procedure and would recommend it. Thirty-three percent of the patients did not report satisfaction and would not recommend the procedure or were unsure whether to recommend it. When the objective patient evaluation of the treatment of osteoarthritic knee joints with freshly isolated SVF cells was sorted based by the stage of arthritis and the use of PRP, it became obvious that the patients with stage 4 osteoarthritis that were treated with the SVF without the addition of PRP showed the lowest scoring of their procedure as 100% of them stated that they were dissatisfied.

### 3.5. Side Effects of the SVF Treatment

No serious adverse events or unwanted side effects related to the SVF treatment were recorded by the physicians or by the patients. Merely some pain-free swelling of the injection site that resolved on its own in the following 24 h was reported by some patients. Some hematomas and muscle sorelike pains that resolved without further interventions were sporadically described for the tissue harvesting site. At no point in time were infections of the tissue harvesting or the treatment site observed.

## 4. Discussion

In this study, we tested the treatment of patients with knee osteoarthritis with SVF cells separated with a novel device. One of the most pressing questions regarding regenerative treatment approaches with adipose tissue-derived SVF is the question of cell dosage. Regarding the treatment of arthritic joints, this question could not be answered conclusively so far. Especially, studies using freshly isolated SVF for the treatment of arthritic joints rarely addressed the applied number of nucleated cells, let alone dose dependency. In a dose escalation trial with autologous laboratory-expanded ASCs, only the treatment with a low dose of ASCs (2 × 10^6^ cells) led to significant improvements in pain level and function, compared with the treatment with medium (10 × 10^6^ cells) and high doses (50 × 10^6^ cells) [20]. Using bone marrow-derived mesenchymal stem/stromal cells (MSCs), Lamo-Espinosa et al. found a more distinct improvement in the high-dose treatment group (100 × 10^6^ cells) compared with the low-dose treatment group (10 × 10^6^ MSC) [23]. Jo et al. observed improvement of the Western Ontario and McMaster Universities Arthritis Index (WOMAC) score only in patients treated with 100 × 10^6^ ASCs compared with those treated with lower treatment dosages (10 × 10^6^ cells and 50 × 10^6^ cells) [24]. In a study using freshly isolated SVF for the treatment of eight patients with osteoarthritic knees, between 4.2 × 10^6^ and 41 × 10^6^ cells were administered to each knee joint, the average being 14.1 × 10^6^. The authors stated that they did not observe a dose-dependent response to the amount of SVF injected [18]. In another study using freshly isolated SVF for the treatment of osteoarthritic knees, the patients were allocated to a high-dose (3 × 10^7^ cells), low-dose (1.5 × 10^7^ cells), or placebo group. In both treatment groups, an improvement of the clinical parameters was observed. In the case of this study, the effects were dose-dependent, with higher dose showing higher effects [25]. In this case series, we did not administer predefined numbers of SVF cells but the maximum number that could be collected from each patient’s tissue, which was on average 7.56 × 10^6^ SVF cells. The injection volumes varied between 7 and 37 mL. This was a result of the need of the fivefold dilution of the pellet extraction volume (described in Section 2.5). Independent of the injection volume, there was never a buildup of pressure in the joint during injection. Using the Pearson correlation coefficient, our data show a low but negative correlation between the number of administered cells and KOOS score improvement (see Table 3): −0.27 at the 3-month follow-up and more distinctly −0.35 at the 12-month follow-up.

This means that the lower is the number of administered cells, the better is the KOOS score improvement. It needs to be noted that, in the studies cited here, laboratory-extended cells were used for the treatments. This way, it is more feasible to produce a cell product with a defined cell number. These preparations are not the same product as a freshly isolated autologous SVF. Cultured ASCs constitute a fairly homogenous cell population. The SVF is a heterogeneous mixture of cells that can exert different regenerative functions. Adipose tissue-derived stem/stromal cells can contribute to cartilage regeneration by tissue-specific differentiation, secretion of the extracellular matrix, and secretion of various immune-modulating factors [5,26,27]. Besides ASCs, the SVF contains additional cell types that can themselves contribute to tissue regeneration. Macrophages possess tissue-specific differentiation potential; regulatory T cells and macrophages secrete immunomodulatory factors and cytokines; and fibroblasts secrete extracellular matrix components that have a positive influence on cell adhesion, migration, and cell matrix interactions (reviewed in [28]). Some studies and reviews even suggest that, due to its heterogeneity, the SVF might be more potent in its regenerative capacity than cultured ASCs [28,29,30]. Due to the involvement of differing cell products, the comparability of the results of the studies is limited. As the number of patients in this case series and in the cited studies was low, no conclusive statement can be made on the dose of cells necessary to treat an arthritic joint. But our results and some of the literature do perhaps imply that a higher cell dosage is not necessarily a more effective one. More investigations are needed to elucidate this aspect.

The pre- and postoperative imaging revealed two phenomena that could be observed in the SVF-treated joints. The first was a rearrangement of the cartilage structure and alleviation of effusions after 16 months of the SVF treatment; this could represent cartilage regeneration. From the images, it cannot be concluded which type of cartilage was formed. It can be considered a successful regeneration of the articular cartilage if a formation of hyaline cartilage can be found on the surface of the joints. Previous studies have shown the de novo formation of hyaline-like cartilage after treatment with ASCs and umbilical cord blood-derived MSCs [24,25]. The second occurrence that could be observed on the MRI images was a widening of the joint space 14 months after the SVF treatment. This was documented before by Michalek et al., who observed a broadening of joint spaces 6–12 months after autologous adipose tissue-derived SVF treatment [8]. Lamo-Espinosa et al., who used bone marrow-derived MSCs, did not observe a widening of the joint space in their treatment groups. Rather, they observed a decreasing joint space in the placebo control group that appeared to have been intercepted by the cell treatment [23]. Not in all patients could changes be detected when comparing pre- and postoperative images. Nonetheless, even patients without visual regeneration benefited from the treatments, experiencing notable pain relief.

In this case series, the patients with grade 4 osteoarthritis that were treated with the SVF without the addition of PRP showed the highest rate of dissatisfaction with the outcome of their procedure. A parallel can be drawn here to the progression of the KOOS scores. After 12 months, the improvements of the scores were higher than 8 points in the treatment groups, grade 3 without PRP (+34.5%), grade 3 with PRP (+9.7%), and grade 4 with PRP (+28.8%), and thus met the suggested criterion of minimal perceptible clinical improvement [26]. For the treatment group grade 4 without PRP, no improvement of their KOOS score was recorded; it dropped by 7.7% after 12 months. This was also reflected by the calculations of the correlation between the parameter “OA grade” and the improvement of the KOOS score at the 3- and 12-month follow-up time points (see Table 3). With a value of −0.10, we saw a low but negative correlation after 3 months that became more distinct after 12 months with an r value of −0.23. This negative correlation implies that the higher is the grade of the osteoarthritis, the lower is the KOOS score improvement. These observations imply that, overall, patients with lower-grade osteoarthritis may have a higher probability of benefitting from the treatment with the SVF. Tran et al. found a better improvement after intra-articular SVF injection in patients with grade 3 OA compared with patients with grade 2 OA [16]. In that case, the patient group with higher-grade OA benefited most from the treatment. On the other hand, one could say that, in this case series, as well as in the examinations done by Tran et al., the patient group with grade 3 OA benefited most from the treatment. In a 2016 study by Nguyen et al., it was observed that both patient groups (grade 2 and 3 OA) benefited from the SVF treatment compared with a placebo control group, but the group of the grade 2 OA patients displayed more distinct improvements in their WOMAC and Lysholm scores [15]. Although, according to our results and the literature, it appears more likely that lower-grade OA patients benefit more from the SVF treatment, it is evident that, in this study as well as in the other studies cited, the patient numbers were too low for a conclusive verdict. This is substantiated by the fact that, in contrast to the treatment group grade 4 without PRP, the treatment group with PRP had the highest subjective evaluation rates. This matter, as well as the benefit of the addition of PRP to the SVF, will need to be examined further.

A negative correlation with the age of the treated patient and the improvement of the KOOS score was observed in this case series. The *r* value of −0.34 at the 12-month follow-up might imply that there are lower improvements of the KOOS scores with the increase of the age of the patients.

One big limitation of this case series, as has been mentioned before, is the limited number of patients. Further examinations will be needed to elucidate if patients with lower-grade OA do indeed receive a higher benefit from the SVF treatment and if the benefit of the treatment indeed decreases with age. Also, the question of whether the addition of PRP to the treatment does add an advantage has not been conclusively resolved. As such, the authors understand their work as the establishment of more specific questions that can now be further investigated.

## 5. Conclusions

On the basis of the data collected from this case series, we can conclude that the treatment of arthritic knee joints with the intra-articular injection of autologous adipose tissue-derived SVF is a safe procedure with clinical benefit for patients. The “psychological path” to an irreversible prosthetic joint replacement is easier for patients when they know that all other minimally invasive therapy options have been exhausted. The SVF treatment may provide alleviation of the OA as well as a gain in time before joint replacement surgery becomes necessary, thereby avoiding multiple changes of prostheses. It can also represent an alternative for patients that might not have the physical fitness to endure a lengthy invasive procedure like a joint replacement. Even if the treatment of osteoarthritis of large joints with the SVF from the adipose tissue fails, a conservative surgical treatment with an endoprosthesis can still be followed. This is not possible the other way round. Thus, prior to conducting a total joint arthroplasty, the treatment of arthritic knee joints with intra-articular injection of autologous adipose tissue-derived SVF should be considered as a regenerative treatment option.

## Figures and Tables

**Table 1:** Patient demographics.

Treatment Group	No. of Patients	Age	Gender		BMI
			Female	Male	
Total	12	61 (51–80)	5 (42%)	7 (58%)	26.4 (20.0–35.3)
Grade 3 SVF	3	55 (51–58)	1	2	28.6 (24.7–35.3)
Grade 3 SVF + PRP	3	57 (51–66)	1	2	26.4 (20.7–34.6)
Grade 4 SVF	3	67 (54–80)	1	2	24.2 (20.0–29.0)
Grade 4 SVF + PRP	3	64 (52–75)	2	1	26.4 (21.6–29.0)

SVF, stromal vascular fraction; PRP, platelet-rich plasma; BMI, body mass index.

**Table 2:** Cell numbers and injection volumes for the treatments of knee osteoarthritis of one single knee joint with freshly isolated SVF from the adipose tissue.

Treatment Group		Injection Vol. (mL)	Cell Number (× 10^6^)
Grade 3 SVF	Patient 1.1	9	4.24
	Patient 1.2	10	4.96
	Patient 1.3	7	17.2
Grade 3 SVF + PRP	Patient 2.1	37	5.81
	Patient 2.2	24	7.53
	Patient 2.3	20	7.20
Grade 4 SVF	Patient 3.1	9.5	8.19
	Patient 3.2	9.5	3.57
	Patient 3.3	18	9.63
Grade 4 SVF + PRP	Patient 4.1	19	6.84
	Patient 4.2	18.5	10.2
	Patient 4.3	19	5.34

SVF, stromal vascular fraction; PRP, platelet-rich plasma; BMI, body mass index.

**Table 3:** Pearson correlation coefficient of age, BMI, injected cell number, and grade of osteoarthritis in relation to the progression of the KOOS scores at the respective follow-up time points of 1, 3, 6, and 12 months after the treatment of knee osteoarthritis with freshly isolated SVF from the adipose tissue.

Pearson Correlation Coefficient (*r*)	Injected Cell Number	OA Grade	Age
KOOS progression at 3 months	−0.2744	−0.09964	−0.0947
KOOS progression at 12 months	−0.3500	−0.2346	−0.3428
